# Micro-Structure Engineering in Pd-InO_x_ Catalysts and Mechanism Studies for CO_2_ Hydrogenation to Methanol

**DOI:** 10.3390/molecules29163715

**Published:** 2024-08-06

**Authors:** Fengwang Zhao, Gemeng Liang, Xiaoli Yang, Yang Lei, Fayi Jin, Leilei Xu, Chuanhui Zhang, Wei Jiang, Haoxi Ben, Xingyun Li

**Affiliations:** 1 State Key Laboratory of BioFibers and Eco-Textiles, Institute of Materials for Energy and Environment, College of Materials Science and Engineering, Qingdao University, Qingdao 266071, China; zhaofengwangedu@163.com (F.Z.);; 2 Hubei Key Laboratory of Coal Conversion and New Carbon Materials, School of Chemical Engineering & Advanced Materials, University of Adelaide, Adelaide, SA 5000, Australia; 3 School of Chemistry and Chemical Engineering, Wuhan University of Science and Technology, Wuhan 430081, China; 4 Jiangsu Key Laboratory of Atmospheric Environment Monitoring and Pollution Control, Joint International Research Laboratory of Climate and Environment Change (ILCEC), Collaborative Innovation Centre of the Atmospheric Environment and Equipment Technology, School of Environmental Science and Engineering, Nanjing University of Information Science & Technology, Nanjing 210044, China

**Keywords:** carbon dioxide, indium oxide, defect engineering, intermetallic compounds

## Abstract

Significant interest has emerged for the application of Pd-In_2_O_3_ catalysts as high-performance catalysts for CO_2_ hydrogenation to CH_3_OH. However, precise active site control in these catalysts and understanding their reaction mechanisms remain major challenges. In this investigation, a series of Pd-InO_x_ catalysts were synthesized, revealing three distinct types of active sites: In-O, Pd-O(H)-In, and Pd_2_In_3_. Lower Pd loadings exhibited Pd-O(H)-In sites, while higher loadings resulted in Pd_2_In_3_ intermetallic compounds. These variations impacted catalytic performance, with Pd-O(H)-In catalysts showing heightened activity at lower temperatures due to the enhanced CO_2_ adsorption and H_2_ activation, and Pd_2_In_3_ catalysts performing better at elevated temperatures due to the further enhanced H_2_ activation. In situ DRIFTS studies revealed an alteration in key intermediates from *HCOO over In-O bonds to *COOH over Pd-O(H)-In and Pd_2_In_3_ sites, leading to a shift in the main reaction pathway transition and product distribution. Our findings underscore the importance of active site engineering for optimizing catalytic performance and offer valuable insights for the rational design of efficient CO_2_ conversion catalysts.

## 1. Introduction

The hydrogenation of carbon dioxide (CO_2_) to methanol (CH_3_OH) represents a crucial catalytic process, pivotal in addressing environmental concerns and fostering sustainable chemical production. This process provides a viable solution for utilizing CO_2_, a significant greenhouse gas [1], while also contributing to the production of CH_3_OH, a versatile chemical feedstock with a wide range of industrial applications [2,3]. However, this process presents formidable challenges, considering the ultra-stable C=O bond in CO_2_ [4,5,6]. Meanwhile, the reverse water–gas shift reaction (RWGS), wherein CO_2_ reacts with hydrogen for producing water, redirects the reaction towards the undesired production of carbon monoxide (CO) rather than CH_3_OH.

To address the above issue, indium oxide (In_2_O_3_) based catalysts have recently garnered widespread attention due to their exceptional productivity for CH_3_OH [7]. Nonetheless, pure In_2_O_3_ exhibits insufficient adsorption capacity for reaction gases, leading to suboptimal reaction efficiency [2]. Additionally, the instability of the In_2_O_3_ structure results in a rapid deactivation of catalysts. Consequently, numerous researchers have explored surface modifications of In_2_O_3_ to enhance its activity and stability by altering its active sites. For instance, Liu et al. demonstrated the introduction of new active sites through nitrogen doping, resulting in a great enhancement of reaction activity and catalytic stability of In_2_O_3_ [8]. Recent discoveries have revealed that the interface sites between Pd and In_2_O_3_ catalysts are more active for the activation of H_2_ [9]. Ge et al. reported the notable improvement for CO_2_ hydrogenation to CH_3_OH by coupling highly dispersed Pd nanoparticles with In_2_O_3_, delivering a methanol productivity of 0.89 g_MeOH_·h^−1^·g_cat_^−1^ [10]. However, it is noteworthy that various catalytic sites may co-exist in addition to the Pd-In_2_O_3_ interfaces in the Pd/In_2_O_3_ system. For example, metal Pd might tend to form large particles, exacerbating the competitive RWGS reaction, and leading to the increase in CO byproduct [11].

Meanwhile, Pd can also form alloys with other metals, resulting in the tailored active site for CO_2_ hydrogenation reactions. Bowker et al. reported that ZnO-supported Pd-Zn alloys with optimized particle size and surface structure demonstrated a high performance to activate CO_2_ with CO_2_ conversion of 11% and methanol selectivity of 60%, at conditions of 250 °C and 20 bar [12]. Song et al. used SiO_2_ as support for Pd-Cu alloy particles. With the optimization of Pd/(Pd + Cu) atomic ratios, it was found that Pd(0.34)-Cu/SiO_2_ was most active with CH_3_OH yield of 0.31 µmol·gcat^−1^·s^−1^ [13]. Additionally, Pd-In alloy may be simultaneously created, which may also shine for CO_2_ hydrogenation to CH_3_OH [14]. These complex multi-structures might complicate the reaction route, incubating different intermediates, which makes it difficult to achieve a clear understanding of the reaction mechanism [15,16,17]. Thus, engineering specific active sites and exploring the structure–function relationship is crucial, albeit challenging for the development of advanced Pd/In_2_O_3_catalyst [14,18].

In this study, Pd-InO_x_ catalysts with tailor-orchestrated structures were precisely designed and synthesized. The cryogenic activity has great advantages over existing work. Combined (in situ) characterizations were systematically conducted to shed light on the reaction intermediates and reaction pathways. These findings provide valuable insights into the catalytic mechanism of InO_x_-based catalysts, offering guidance for the rational design and development of catalysts for CO_2_ hydrogenation to CH_3_OH.

## 2. Results and Discussion

### 2.1. Structure Characterization of Pd-InO_x_

**Figure 1:**
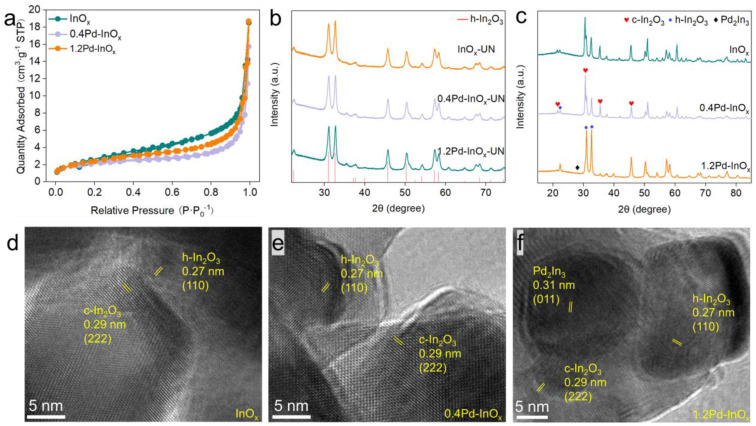
(**a**) N_2_ adsorption–desorption isotherms, XRD patterns of different samples before (**b**) and after (**c**) calcination and reduction. HR-TEM images of (**d**) InO_x_, (**e**) 0.4Pd-InO_x_ and (**f**) 1.2Pd-InO_x_.

The synthesis of InO_x_ with varying Pd contents was achieved using a two-step method. Analysis by ICP-AES revealed the actual Pd content in 0.4Pd-InO_x_ and 1.2Pd-InO_x_ are 0.40 *wt.*% and 1.28 *wt.*%, respectively (Table S1), consistent with the theoretical values. N_2_ adsorption–desorption isotherms in Figure 1a indicate that the specific surface area of all samples is below 11 m^2^·g^−1^, as summarized in Table S1. XRD analysis depicted the presence of the h-In_2_O_3_ phase (JCPDS 04-005-4422) in all InO_x_ and Pd-InO_x_ samples before calcination (Figure 1b). Upon subsequent calcination and reduction of these samples, a coexistence of h-In_2_O_3_ and c-In_2_O_3_ phases (JCPDS 97-001-4388) was observed over pure InO_x_ and 0.4Pd-InO_x_ samples, indicating a crystal transition of In_2_O_3_ (Figure 1c) while only the h-In_2_O_3_ phase was observed in 1.2Pd-InO_x_. Moreover, no diffraction peaks associated with Pd were discernible in the 0.4Pd-InO_x_ sample, indicating highly dispersed Pd on the InO_x_ substrate [19]. In contrast, diffraction peaks corresponding to the Pd_2_In_3_ intermetallic compound were evident (JCPDS 97-064-0231) in the 1.2Pd-InO_x_ sample. The interaction between Pd and In in Pd_2_In_3_ might prevent the crystal transition of h-In_2_O_3_ into the c-In_2_O_3_ phase [20,21].

SEM images revealed a bulky shape for the catalysts, while EDS mapping demonstrated the uniform distribution of In, O and Pd atoms in all xPd-InO_x_ samples (Figures S1 and S2). We further investigated the elemental distribution in the material using STEM-EDX. The distribution of elements in InO_x_ (Figure S3) and 0.4Pd-InO_x_ (Figure S4) is uniform, with no large aggregates observed, which confirms the evenly dispersed state of Pd in 0.4Pd-InO_x_, while for the sample of 1.2Pd-InO_x_, distinct Pd heterogeneous particles are observed (Figure S5). Combined with the XRD results, it can be inferred that Pd_2_In_3_ is formed for the sample of 1.2Pd-InO_x_. (HR-TEM) analysis revealed inter-planar distances of 0.27 nm and 0.29 nm over InO_x_ (Figure 1d) and 0.4Pd-InO_x_ (Figure 1e), corresponding to the (110) facet of h-In_2_O_3_ and (222) facet of c-In_2_O_3_, respectively. These characterizations underscore that InO_x_ and 0.4Pd-InO_x_ constitute a homogeneous structure composed of c-In_2_O_3_ and h-In_2_O_3_. Additionally, a distinct inter-planar distance of 0.31 nm was observed in 1.2Pd-InO_x_, corresponding to the (011) crystal plane of Pd_2_In_3_ (Figure 1f). It suggests that higher loading of Pd leads to some Pd interacting with In to form Pd_2_In_3_ intermetallic compounds. The SAED pattern of Pd-InO_x_ is shown in Figure S6, consisting of polycrystalline diffraction rings. From the innermost to the outermost ring, they are indexed as (011), (−130) of Pd_2_In_3_, respectively. The SAED values are in good agreement with XRD results, further confirming the presence of Pd_2_In_3_ in 1.2Pd-InO_x_.

### 2.2. The Structure–Function Relationship

**Figure 2:**
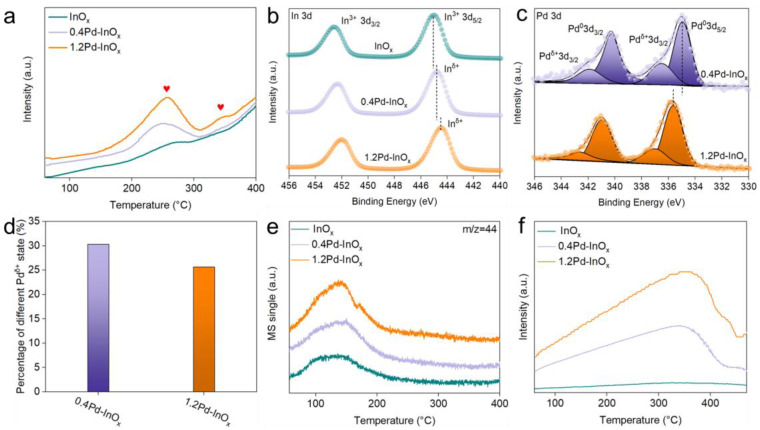
(**a**) H_2_-TPR. The heart symbol represents the peak center. (**b**) Deconvolution of Pd 3d XPS spectra and (**c**) In 3d XPS spectra. (**d**) Percentage of different Pd^δ+^ states. (**e**) CO_2_-TPD and (**f**) H_2_-TPD of different samples.

To further investigate the structural states of these catalysts, H_2_-TPR experiments were conducted on the fresh catalysts. From Figure 2a, peaks at 200–300 °C (as indicated by the red heart) is observed in the curves of all catalysts contributed to the formation of oxygen vacancy on the In_2_O_3_ surface, resulting in the reduced state of InO_x_. Further, compared to InO_x_, the area of this peak becomes stronger over 0.4Pd-InO_x_ and 1.2Pd-InO_x_. It suggests the promoted surface reduction of InO_x_ facilitated by the interaction with Pd [22]. Additionally, a new peak at higher temperatures (300–400 °C) over 1.2Pd-InO_x_ is formed. It may be attributed to the presence of Pd_2_In_3_ intermetallic compounds [23,24], which also suggested that the Pd_2_In_3_ intermetallic compounds significantly benefit the activation of hydrogen.

XPS analysis was carried out to characterize the surface chemical information of the catalysts after reduction. The full XPS spectrum proved clearly the presence of In, O, and Pd as displayed in Figure S7. The In 3d spectra exhibit binding energies consistent with In^3+^ over InO_x_ (Figure 2b) [25,26], while Pd 3d signals could be deconvoluted into four peaks corresponding to Pd^δ+^ and metallic Pd^0^ (Figure 2c) [27,28]. Notably, compared to pure In_2_O_3_, a slight shift towards lower binding energy for In 3d XPS peak was observed for 0.4Pd/InO_x_, indicating a reduced valence state [29]. Previous studies have indicated that the incorporation of Pd in oxides may result in the formation of Pd-O(H)-M bonds [30], while the presence of Pd^δ+^ species in the Pd 3d XPS spectra (Figure 2c) may offer evidence for the existence of Pd-O(H)-In structures for Pd/InO_x_ [31]. It is interesting to find the relative content of Pd^δ+^ decreased as the content of Pd increased to 1.2 *wt.*% (Figure 2d); however, the 3d_5/2_ of In further shifted to a lower binding energy (444.4 eV). This suggests the formation of another chemical bond over 1.2Pd/InO_x_, possibly attributed to the formation of Pd_2_In_3_ intermetallic compound on the surface [18,32]. Thus, Pd species tend to form highly dispersed Pd-O(H)-In sites on the sample surface at a low Pd loading, while partly forming Pd_2_In_3_ sites at high Pd loading, resulting in a surface where Pd-O(H)-In and Pd_2_In_3_ synergistically co-existence. Moreover, the presence of these active sites may alter the adsorption properties and reaction pathway, influencing the catalytic performance of CO_2_ hydrogenation. Further detailed investigations are required to elucidate these effects.

CO_2_-TPD analysis reveals a low-temperature desorption peak for all catalysts, with the desorption temperature remaining unchanged but the peak area slightly increasing with more Pd loadings (Figure 2e) [33]. This phenomenon indicates similar adsorption strength but a slightly enhanced adsorption capacity for Pd/InO_x_ [34,35], which may be due to the beneficial effect of Pd-O(H)-In and Pd_2_In_3_ for the CO_2_ adsorption. It can be observed that after the formation of Pd_2_In_3_, an overlapping CO_2_ desorption peak appears at 171 °C, which may be attributed to the change in the CO_2_ desorption pattern caused by the formation of Pd_2_In_3_ [36]. The H_2_-TPD analysis could demonstrate the catalyst’s capability for dissociating and activating H_2_. On pure InO_x_, there is virtually no desorption peak, indicating a weak adsorption and activation capacity for H_2_ on the pure In-O bonds. As the Pd loading increases, the desorption peak area for xPd-InO_x_ enhances at elevated temperatures (300–400 °C), as depicted in Figure 2f. In comparison, the peak area for 1.2Pd-InO_x_ is greater than that for 0.4Pd-InO_x_. Considering the formation of Pd-O(H)-In and Pd_2_In_3_ active sites within the Pd-InO_x_ catalyst, this finding may corroborate that the Pd-O(H)-In site plays a more significant role in facilitating H_2_ activation at lower temperature [9], whereas Pd_2_In_3_ is more favorable for H_2_ activation at higher temperatures [14]. This is in agreement with the H_2_-TPR results. It is to be mentioned that a minor desorption peak observed at approximately 436 °C for 1.2Pd-InO_x_ may be ascribed to the spillover of H_2_ from Pd_2_In_3_ to the support [37].

### 2.3. Catalytic Performance for CO_2_ Hydrogenation

**Figure 3:**
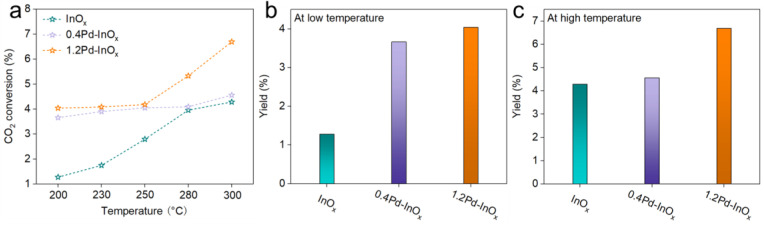
(**a**) CO_2_ conversion. Methanol yield of different samples at (**b**) 200 °C and (**c**) 300 °C. Reaction condition: 5 MPa, CO_2_/H_2_ = 1/3, and 3000 mL·g_cat_^−1^·h^−1^.

Under reaction conditions of 5 MPa and a gas hourly space velocity (WHSV) of 3000 mL·gcat^−1^·h^−1^, CO_2_ hydrogenation experiments were conducted. As shown in Figure 3a and Figure S8, the catalytic performance varies significantly among these three catalysts. At 200 °C, the CO_2_ conversion rate of InO_x_ is only 1.28%. In contrast, for 0.4Pd-InO_x_ and 1.2Pd-InO_x_, the CO_2_ conversion rates at 200 °C are 3.66% and 4.04%, respectively. It implies the promoted role of Pd-O(H)-In sites and Pd_2_In_3_ sites in CO_2_ conversion. Interestingly, the change in the CO_2_ conversion rate with the increase in reaction temperature differed obviously for these catalysts. For InO_x_, the CO_2_ conversion rate improved from 1.28% at 200 °C to 4.20% at 300 °C, while 0.4Pd-InO_x_ demonstrates a slight CO_2_ conversion rate increase to 4.56% at 300 °C. Prominently, for 1.2Pd-InO_x_, its CO_2_ conversion rate increased notably at elevated temperatures, reaching 6.69% at 300 °C. These differences are attributed to the varied active sites over these catalysts [5,33].

To obtain a more comprehensive understanding of the reaction trend, the yields of CH_3_OH were calculated. According to the curves, the yield change was divided into the low-temperature region (200–250 °C) and the high-temperature region (250–300 °C). The InO_x_ catalyst, with limited activation capability for H_2_, yields less than 2% in the lower-temperature region (Figure 3b). With the increase in Pd loading, the yields increase to over 3% for 0.4Pd/InO_x_ and 1.2Pd/InO_x_ catalysts. This might be attributed to the enhanced CO_2_ and H_2_ adsorption capability, especially for the H_2_ adsorption capability at lower temperatures due to the formation of Pd-O(H)-In sites. However, in the higher-temperature region, the yield of 1.2Pd-InO_x_ reached over 6%, much higher than the yields of InO_x_ (4.25%) and 0.4Pd-InO_x_ (4.28%) (Figure 3c). The more favorable reaction of 1.2Pd-InO_x_ at high temperatures may be due to the formation of Pd_2_In_3_ which greatly enhances its ability to adsorb and dissociate H_2_ at high temperatures. These phenomena illustrate that the varied active sites might play different roles in the reaction intermediates and pathways, which will be further discussed. We provide a comparison of the performance of Pd-based catalysts in Table S2.

### 2.4. Reaction Mechanisms

The activation pathways for CO_2_ and H_2_ to produce CH_3_OH were well established by the previous reports [38]. Initially, CO_2_ adsorbs onto the sample surface, forming *CO_2_, which then reacts with lattice oxygen (O_lattice_) or dissociated hydrogen (*H) to produce either *CO_3_^2−^ or *HCO_3_^−^. Subsequently, CH_3_OH is generated through two possible pathways. The first pathway involves the formation of *CO intermediate, generated via the RWGS routes involving carboxyl (*COOH) species. This intermediate is then hydrogenated to produce CH_3_OH. The second pathway entails the formation of formate (*HCOO) intermediates through the hydrogenation of CO_2_, ultimately leading to the production of CH_3_OH through C-O bond cleavage and *HCO or *H_2_CO intermediates (referred to as the formate pathway).

**Figure 4:**
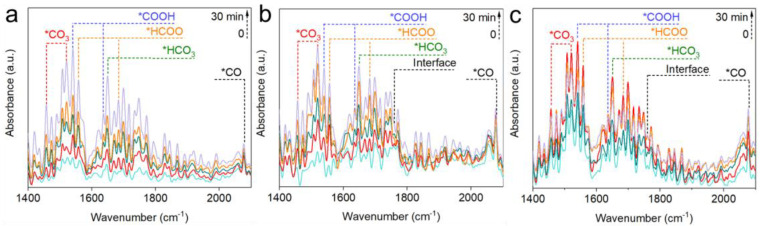
(**a**) In situ DRIFT spectra of InO_x_, (**b**) 0.4Pd-InO_x_, and (**c**) 1.2Pd-InO_x_. Reaction conditions: 200 °C, CO_2_/H_2_ = 1/3, 10 mL·min^−1^, and 0.1 MPa.

**Figure 5:**
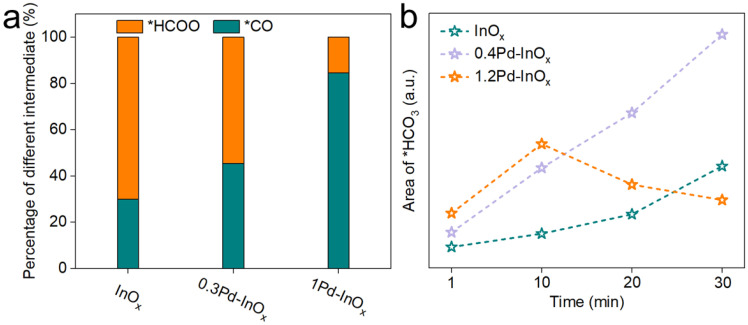
(**a**) Percentage of different intermediates over the xPd/InO_x_ catalysts calculated by in situ DRIFT. (**b**) The trend of in situ DRIFT peak area changes for *HCO_3_^−^.

To elucidate the effect of varied active sites on intermediate evolution, in situ DRIFTS studies were conducted on InO_x_ (Figure 4a), 0.4Pd-InO_x_ (Figure 4b), and 1.2Pd-InO_x_ (Figure 4c). *CO_3_^2−^ at 1456 and 1520 cm^−1^ and *HCO_3_^−^ at 1650 cm^−1^ are observed on InO_x_ [39,40]. Subsequently, formate at 1559 and 1683 cm^−1^, as well as *COOH at 1540 and 1637 cm^−1^, are detected with time on stream [31,38], along with a small amount of *CO at 2077 cm^−1^ [8,41]. In comparison to InO_x_, the initial area of CO_3_^2−^ and HCO_3_^−^ is significantly higher for 0.4Pd-InO_x_ and 1.2Pd-InO_x_, which may be due to the enhancement of CO_2_ adsorption caused by the presence of Pd-O(H)-In sites. Furthermore, a new peak corresponding to the adsorption of *COOH at the interface appears at 1758 cm^−1^, and the proportion of *CO and *COOH intermediates increases, as shown in Figure·5a and Table S3. This suggests that the appearance of Pd-O(H)-In sites alters the main reaction pathway transition, initiating the conversion of the key intermediate in CH_3_OH synthesis from *HCOO to *COOH and *CO [15,29]. This likely occurs because the formation of Pd-O(H)-In modulates the catalyst’s activation capability concerning *H [42]. H_2_ undergoes rapid activation at Pd-O(H)-In sites, subsequently converting to *COOH intermediates on the surface [43]. For 1.2Pd-InO_x_ that contained Pd_2_In_3_ sites, the peak intensity of *HCO_3_^−^ initially increases and then decreases. It indicates that the modulation of H_2_ adsorption and dissociation capability by Pd_2_In_3_ renders the catalyst more favorable for the transition from *HCO_3_^−^ to other intermediates (Figure 5b).

**Figure 6:**
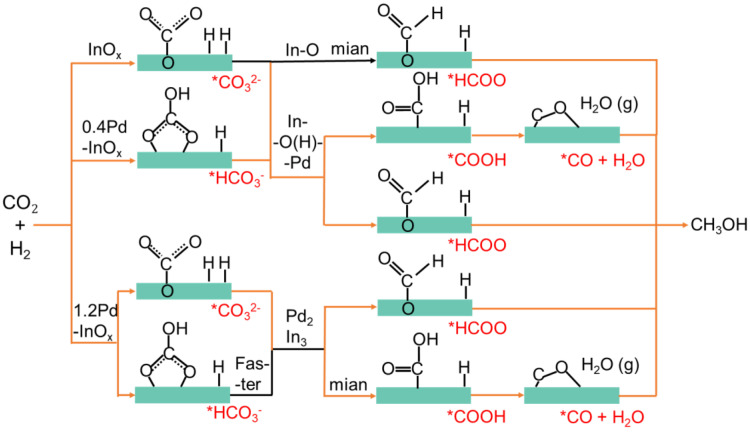
Possible reaction pathways corresponding to different active sites.

Based on the phenomenon, distinct active sites might induce different reaction pathways, as summarized in Figure 6 [24]. On the InO_x_ surface, CO_2_ and H_2_ were activated on In-O, generating small amounts of *CO_3_^2−^ and *HCO_3_^−^, which are then hydrogenated to form *HCOO, leading to CH_3_OH production primarily through the formate pathway [2,44]. With the formation of Pd-O(H)-In interfacial sites on 0.4Pd-InO_x_, the initial peak area of *HCO_3_^−^ and *CO_3_^2−^ increases. Subsequently, a larger number of *COOH and *CO intermediates are formed as H_2_ is more readily activated over Pd-O(H)-In sites, leading to CH_3_OH synthesis through the reverse water–gas pathway [45]. After the formation of Pd_2_In_3_ sites over 1.2Pd-InO_x_, H_2_ becomes more accessible to be activated compared to when only Pd-O(H)-In sites are present [42]. It could be further observed that the content of *HCO_3_^−^ intermediates shows a trend of initially increasing and then decreasing, which aids in the continued conversion of *HCO_3_^−^ to *COOH and *CO, further hydrogenating to produce CH_3_OH.

## 3. Experimental Section

### 3.1. Preparation of InO_x_ Catalysts

Firstly, 2.0 g of indium nitrate (99.99%, Macklin, Shanghai, China) was dissolved in a solution comprising 30 mL deionized (DI) water and 60 mL ethanol (AR, Sinopharm, Shanghai, China). Subsequently, 0.1 mL of nitric acid (AR, Sinopharm, Shanghai, China) was added. After complete dissolution, the obtained homogeneous solution was transferred into a Teflon-lined autoclave with a capacity of 200 mL and placed in an oven at 150 °C for 12 h. Upon naturally cooling down to room temperature, the resulting product was collected by centrifugation, washed with plenty of DI water and ethanol, and then dried at 60 °C overnight to obtain the InO_x_-UN. The InO_x_-UN was calcinated at 450 °C for 3 h in air to obtain the InO_x_. Before characterizations and the catalytic applications, InO_x_ was reduced by H_2_ at 380 °C for 120 min, except for H_2_-TPR.

### 3.2. Preparation of Pd-InO_x_ Catalysts

The Pd-InO_x_ samples were synthesized using the impregnation method. The palladium nitrate solution (Pd 18.09 *wt.*% in nitric acid, Macklin, Shanghai, China) was diluted with DI water to a concentration of 5.04 g·L^−1^, designated as solution A. Subsequently, 0.8 g of InO_x_-UN (the precursor before reduction and roasting) was dispersed in 100 mL of ethanol under stirring, followed by the addition of 0.48 mL of solution A, which was stirred to dry at 70 °C. The resulting product was further dried overnight at 60 °C to obtain the 0.4Pd-InO_x_-UN. The samples were calcinated at 450 °C for 3 h in air, denoted as 0.4Pd-InO_x_ (the number represented the weight percentage of Pd). 1.2Pd-InO_x_ was also prepared with a similar procedure to that of 0.4 Pd-InO_x_ but adding 1.60 mL of palladium nitrate solution. Before characterizations and the catalytic applications, the catalysts were reduced by H_2_ at 380 °C for 120 min, except for H_2_-TPR.

### 3.3. Catalyst Characterizations

The Inductively coupled plasma-atomic emission spectroscopy (ICP-AES) measurements were carried out on an Avio 200 instrument (Thermo Electron Corporation, Massachusetts, USA). N_2_ adsorption–desorption isotherms were measured on a Micromeritics ASAP 2460 (Micromeritics, Shanghai, China) at -196 °C. All the samples were outgassed at 150 °C overnight before the measurement. The specific surface area was calculated using the Brunauer-Emmett-Teller (BET) method. Scanning and transmission analytical electron microscopy (STEM-EDX) was performed using the FEI Talos F200X (FEI, Hillsboro, UAS). High-resolution transmission electron microscopy (HR-TEM) and selected area electron diffraction (SAED) was performed using the JEM 2100F (JEOL, Japan). X-ray diffraction (XRD) patterns were collected on a Japanese Science Ultima IV (Rigaku Corporation, Japan) diffractometer equipped with a Cu Kα radiation source, operated at 40 kV and 40 mA. The data were collected at a scan speed of 5°·min^−1^ in the 2θ range of 15–85°.

X-ray photoelectron spectroscopy (XPS) experiments were carried out on a Thermo Scientific ESCALAB Xi+ XPS instrument (Thermo, Massachusetts, USA). Spectra of In 3d, C 1s, and Pd 3d were obtained, which were calibrated with the C 1s peak at 284.8 eV. To avoid the influence of air for the XPS test, the sample was protected by Ar gas first and then was vacuum packaged before transferring to the chamber of the XPS equipment. Hydrogen temperature-programmed reduction (H_2_-TPR) was performed on a ChemBET Pulsar (Quantachrome, Florida, USA). Typically, 50 mg of sample was placed in a U-pipe and purged with an argon flow (30 mL·min^−1^) at 260 °C for 60 min. The temperature was then decreased to 50 °C. Meanwhile, the gas flow was switched to a 10% H_2_/Ar mixture (30 mL·min^−1^) and the temperature was increased from 50 to 400 °C at a rate of 10 °C·min^−1^. The effluent gas was monitored in real time via a thermal conductivity detector (TCD). CO_2_ temperature-programmed desorption (CO_2_-TPD-MS) was performed on a lab-made reactor, and the outlet gases were analyzed by mass spectrum (HPR-20 EGA, HIDEN, Warrington, UK). In particular, the catalysts were flushed with He gas flow (30 mL·min^−1^) at room temperature for 30 min. Next, the catalysts were saturated with 10% CO_2_/He (30 mL·min^−1^) at room temperature for 30 min, followed by purging with He flow (30 mL·min^−1^) for 30 min. Then, the CO_2_-TPD experiment was measured from room temperature to 400 °C with a ramping rate of 10 °C·min^−1^ under He gas flow (30 mL·min^−1^). H_2_ temperature-programmed desorption (H_2_-TPD) was also performed on a ChemBET Pulsar (Quantachrome, FL, USA). The catalysts were flushed with He gas flow (30 mL·min^−1^) at room temperature for 30 min. Next, the catalysts were saturated with 10% H_2_/He (30 mL·min^−1^) at room temperature for 30 min, followed by purging with He flow (30 mL·min^−1^) for 30 min. Then, the H_2_-TPD experiment was measured from room temperature to 450 °C with a ramping rate of 10 °C·min^−1^ under He gas flow (30 mL·min^−1^).

In situ diffuse reflectance infrared Fourier transform spectroscopy (in situ DRIFTS) spectra were collected using a Thermo IS10 FTIR (Massachusetts, USA) spectrometer with a mercury cadmium telluride (MCT) detector cooled with liquid nitrogen. The CO_2_ and H_2_ treatment on the xPd-InO_x_ catalyst was investigated at 200 °C. Before the measurement, 30 mg of the sample was pretreated at 380 °C for 30 min under 10% H_2_/Ar mixed gas. Then, the gas was changed to Ar for 30 min. After background acquisition, the reaction gas with H_2_/CO_2_/Ar = 69/23/8 was introduced into the chamber. All DRIFTS results were analyzed by using OPUS software (OMMIC 9.2).

### 3.4. Catalytic Performance Evaluation

The catalytic performance of CO_2_ hydrogenation was evaluated using a vertical fixed-bed reactor. Before the reaction, the catalyst was in situ reduced by H_2_ (99.999%) at 380 °C for 120 min. After cooling to room temperature, the feed reactants (H_2_/CO_2_/Ar = 69/23/8) were introduced at a weight hourly velocity (WHSV) of 3000 mL·gcat^−1^·h^−1^ under 5 MPa with the reaction temperature ranging from 200 to 300 °C. Effluent analysis was performed by an online gas chromatograph (Agilent 8890, Beijing, China) equipped with a flame-ionized detector (FID) and a thermal conductivity detector (TCD). To prevent CH_3_OH condensation, all lines and valves post the reactor were maintained at 170 °C.

## 4. Conclusions

In this study, the investigation focused on Pd-containing InO_x_ catalysts for CO_2_ hydrogenation, aiming to uncover the structure–function relationships. A comprehensive suite of characterization techniques revealed distinct structural disparities among the catalysts. Lower Pd loadings exhibited Pd-O(H)-In sites, while higher loadings resulted in Pd_2_In_3_ intermetallic compounds. Catalysts with Pd-O(H)-In sites exhibit heightened activity at lower temperatures, attributed to enhanced CO_2_ adsorption and H_2_ activation capabilities. Conversely, catalysts featuring Pd_2_In_3_ showed enhanced performance at elevated temperatures, facilitated by the strengthened H_2_ activation. In situ DRIFTS studies revealed a shift in the main reaction pathway, altering key intermediates from *HCOO over In-O bonds to *COOH over Pd-O(H)-In and Pd_2_In_3_ sites. Moreover, Pd_2_In_3_ significantly enhanced the conversion capability of *HCO_3_^−^. The alteration led to a change in product distribution. These findings contribute to the understanding of InO_x_-based catalysts and offer insights into the design of catalysts for CO_2_ hydrogenation to CH_3_OH.

## Data Availability

Data are contained within the article and Supplementary Materials.

## Supplementary Materials

The following supporting information can be downloaded at: https://www.mdpi.com/article/10.3390/molecules29163715/s1, Figure S1: SEM image and EDS elemental mapping of InO_x_; Figure S2: SEM images and EDS elemental mapping of (a) 0.4Pd-InO_x_ and (b) 1.2Pd-InO_x_; Figure S3: STEM-EDX image of InO_x_; Figure S4: STEM-EDX image of 0.4Pd-InO_x_; Figure S5: STEM-EDX image of 1.2Pd-InO_x_; Figure S6: SAED patterns showing polycrystalline diffraction rings acquired of 1.2Pd-InO_x_; Figure S7: XPS full spectrum of different samples; Figure S8: CH_3_OH selectivity versus temperature. Reaction condition: 5 MPa, CO_2_/H_2_ = 1/3, 3000 mL·gcat^−1^·h^−1^; Table S1: Composition and porous properties of the samples with different Pd contents; Table S2: Comparison of catalytic performance between Pd-InO_x_ and other representative catalysts; Table S3: The assignment of in-situ DRIFT peaks. references [10,12,13,17,46,47,48,49,50] are cited in the Supplementary Materials.
