# Supplementary material for: Micro-Structure Engineering in Pd-InOx Catalysts and Mechanism Studies for CO2 Hydrogenation to Methanol
Source: Molecules. 2024 Aug 6;29(16):3715. doi: 10.3390/molecules29163715 (PMC11357378; doi:10.3390/molecules29163715)
Supplement: Supplementary file 1 [file molecules-29-03715-s001.zip › molecules-3058641-supplementary.pdf]

Supporting Information

Micro−Structure Engineering in Pd−InO_x Catalysts and Mechanism Studies for CO_2 Hydrogenation to Methanol

Fengwang Zhao [1,†], Gemeng Liang [3,†], Xiaoli Yang [1,*], Yang Lei [2], Fayi Jin [1], Leilei Xu [4], Chuanhui Zhang [1], Wei Jiang [1], Haoxi Ben [1,*] and Xingyun Li [1,*]

1 State Key Laboratory of BioFibers and Eco−Textiles, Institute of Materials for Energy and Environment, College of Materials Science and Engineering, Qingdao University, Qingdao 266071, China; zhaofengwangedu@163.com (F.Z.)

2 Hubei Key Laboratory of Coal Conversion and New Carbon Materials, School of Chemistry and Chemical Engineering, Wuhan University of Science and Technology, Wuhan 430081, China

3 School of Chemical Engineering & Advanced Materials, University of Adelaide, Adelaide, SA 5000, Australia

4 Jiangsu Key Laboratory of Atmospheric Environment Monitoring and Pollution Control, Joint International Research Laboratory of Climate and Environment Change (ILCEC), Collaborative Innovation Centre of the Atmospheric Environment and Equipment Technology, School of Environmental Science and Engineering, Nanjing University of Information Science & Technology, Nanjing 210044, China; leileixu88@gmail.com

* Correspondence: xlyang@qdu.edu.cn (X.Y.); benhaoxi@qdu.edu.cn (H.B.); xingyun_2008@qdu.edu.cn (X.L.)

† These authors contribute equally to this manuscript.

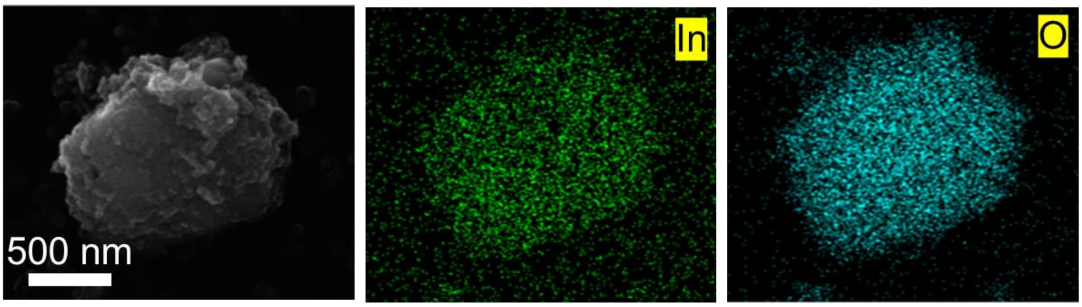

Figure S1. SEM image and EDS elemental mapping of InO_x.

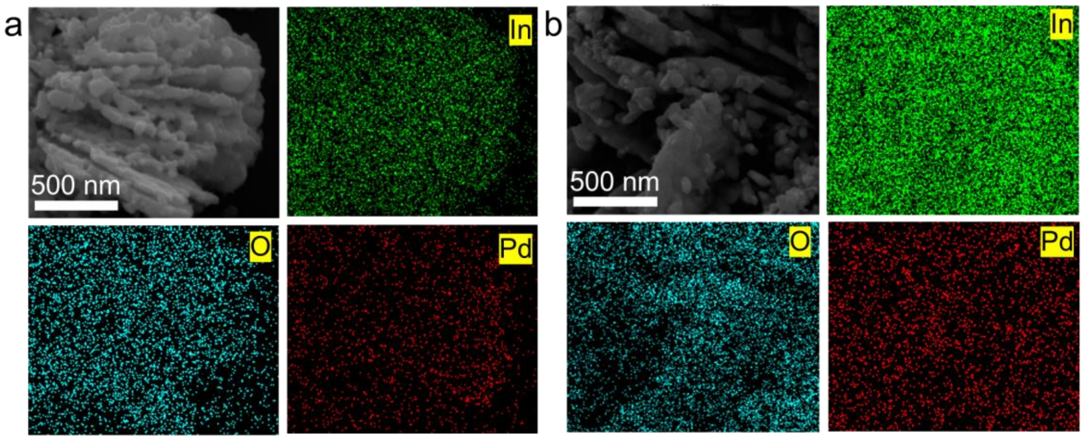

Figure S2. SEM images and EDS elemental mapping of (a) 0.4Pd−InO_x and (b) 1.2Pd−InO_x.

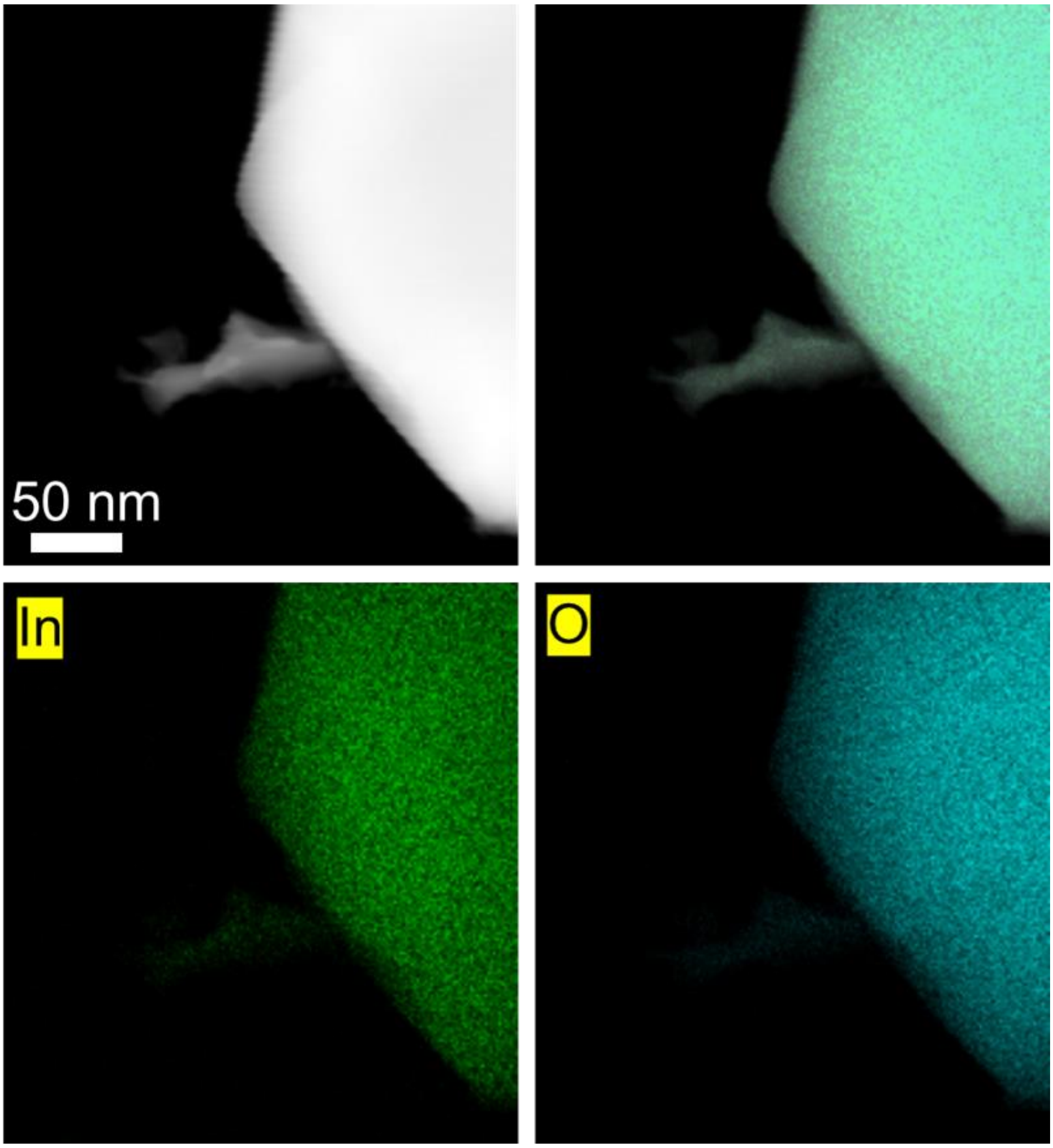

Figure S3. STEM−EDX image of InO_x.

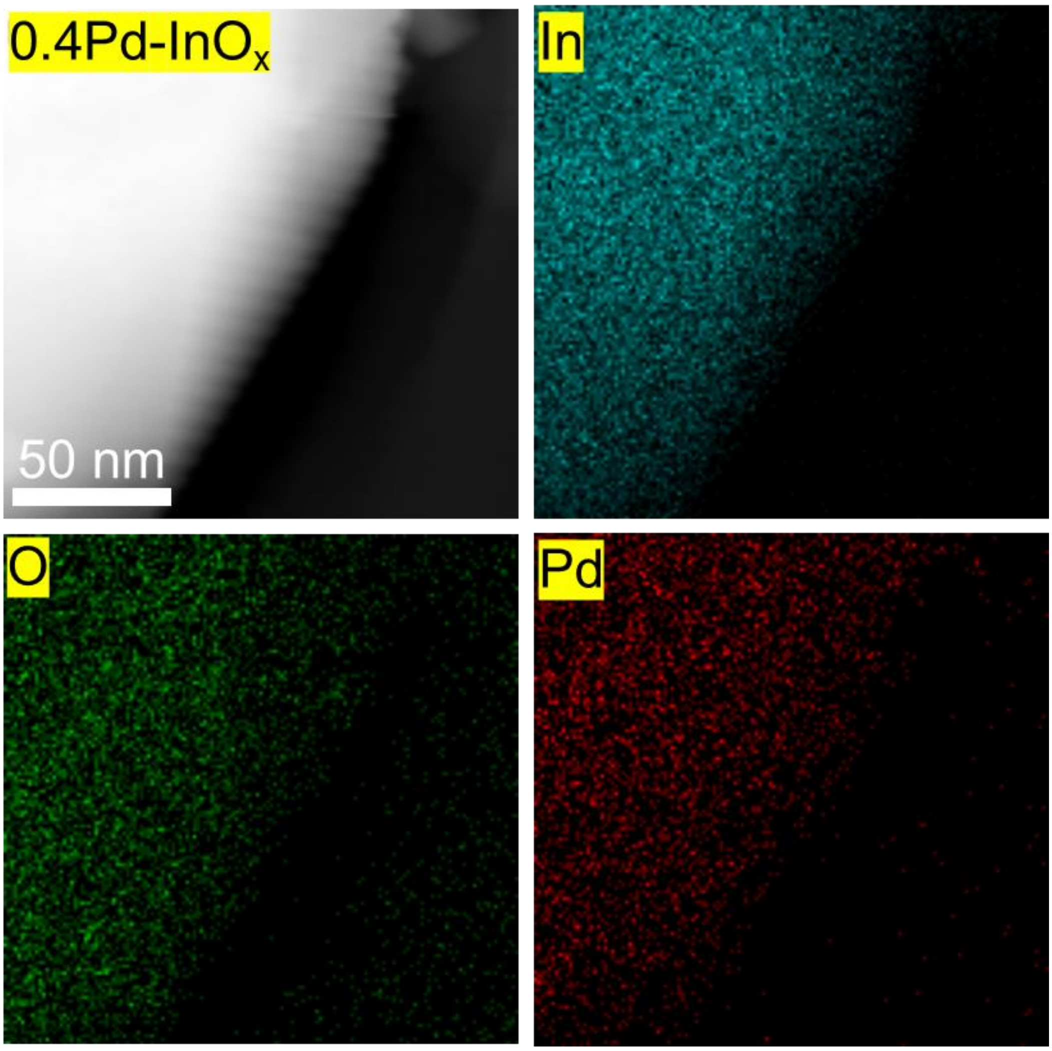

Figure S4. STEM−EDX image of 0.4Pd−InO_x.

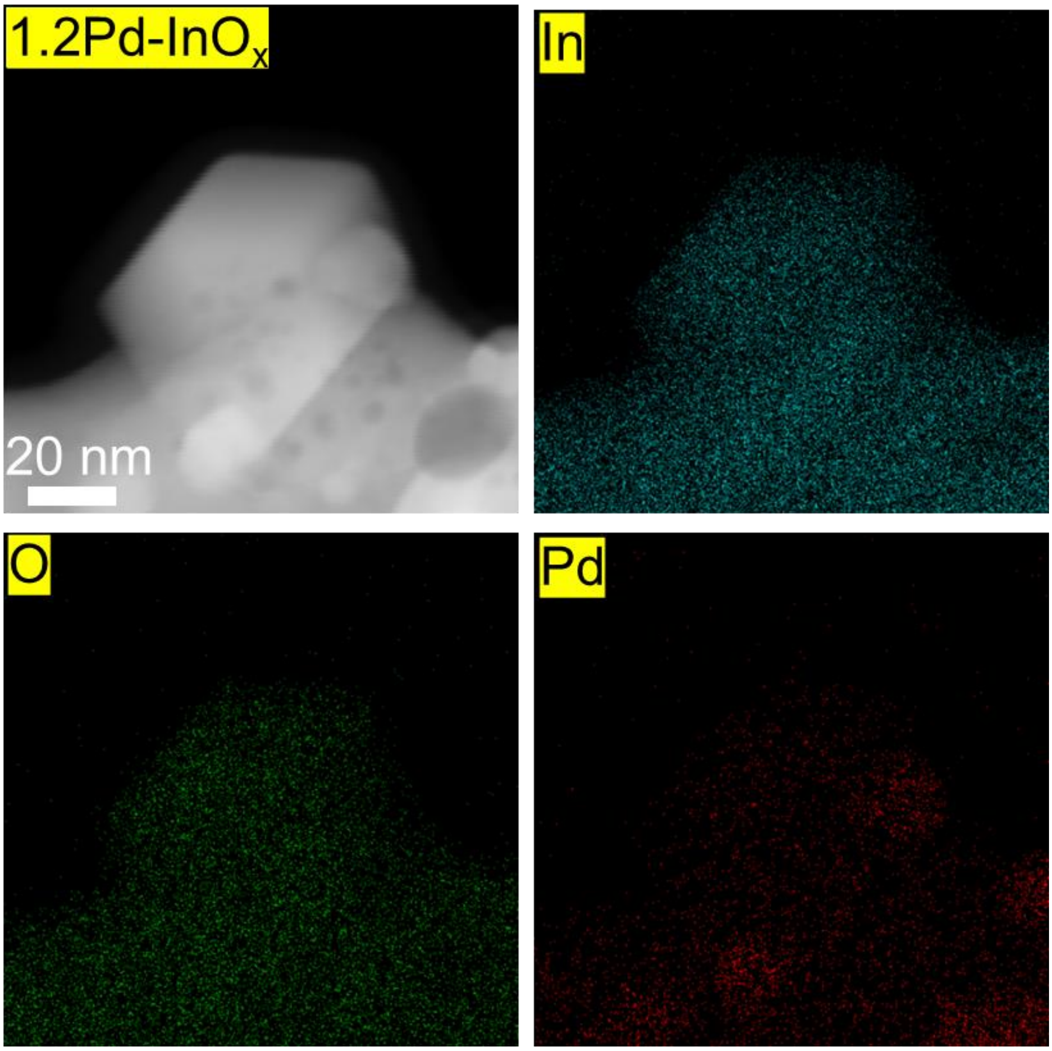

Figure S5. STEM−EDX image of 1.2Pd−InO_x.

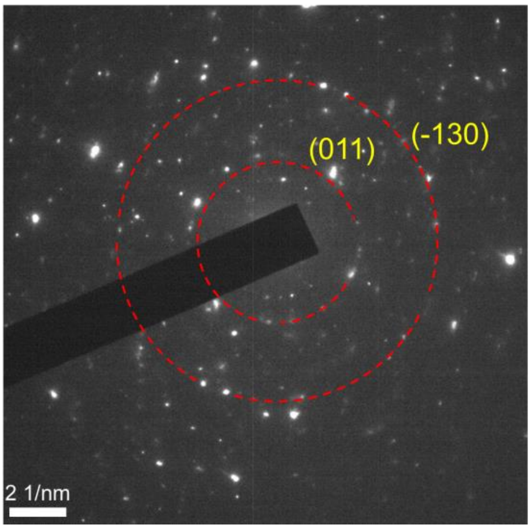

Figure S6. SAED patterns showing polycrystalline diffraction rings acquired of 1.2Pd−InO_x.

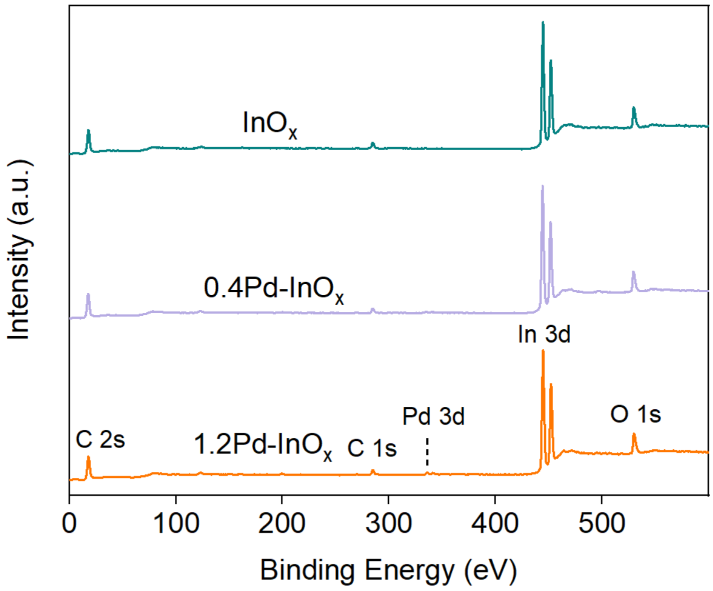

Figure S7. XPS full spectrum of different samples.

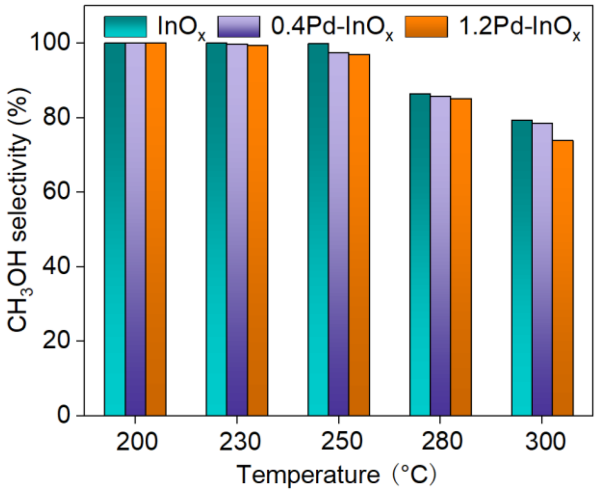

Figure S8. CH_3OH selectivity versus temperature. Reaction condition: 5 MPa, CO_2/H_2 = 1/3, 3000 mL·g_{cat}^{-1}·h^{-1}.

Table S1. Composition and porous properties of the samples with different Pd contents.

Catalysts	Pd content (*wt.* %)[a]	S_{BET} ($m^2 \cdot g^{-1}$)	V_{pore} ($10^{-2} \cdot cm^3 \cdot g^{-1}$)[b]	D_{pore} (nm)[c]
InO_x	–	10.3	2.1	1.0
0.4Pd−InO_x	0.40	7.8	2.3	1.5
1.2Pd−InO_x	1.28	8.9	2.7	1.5

[a]Pd content determined by ICP−AES.

[b]Total pore volume determined at $P/P_0 = 0.99$.

[c]Pore diameter calculated by BJH method.

Table S2. Comparison of catalytic performance between Pd−InO_x and other representative catalysts.

Catalysts	H_2/CO_2 ratio	Pressure (Mpa)	Temperature (℃)	WHSV ($mL \cdot g_{cat}^{-1} \cdot h^{-1}$)	Yield (%)	References
1.2Pd−InO_x	3:1	0.1	200	3000	3.66	**This Work**
2% Pd 300 ℃	3:1	3	200	3600	1.93	[1]
Pd−P/In_2O_3	4:1	5	200	21000	0.8	[2]
Pd/CeO_2−NPH	3:1	5	200	6000	2.37	[3]
PdZn/ZnO	3:1	2	200	−	1.29	[4]
Pd/TiO_2	−	2	230	3600	1.52	[5]
Pd/γ−Al_2O_3	4:1	4	250	9000	0.06	[6]
CoInLDH−350H_2	3:1	3	220	7500	3.1	[7]
PdZnO−0.39Al	3:1	3	250	6000	7.44	[8]
Pd(0.34)−Cu/SiO_2	−	4.1	250	3600	3.93	[9]

1. Xu J.; Su X.; Liu X.; Pan X.; Pei G.; Huang Y.; Wang X.; Zhang T.; Geng H. Methanol synthesis from CO_2 and H_2 over Pd/ZnO/Al_2O_3: Catalyst structure dependence of methanol selectivity. *Applied Catalysis A: General* **2016**, 514, 51−59.
2. Rui N.; Wang Z.; Sun K.; Ye J.; Ge Q.; Liu C.−j. CO_2 hydrogenation to methanol over Pd/In_2O_3: effects of Pd and oxygen vacancy. *Applied Catalysis B: Environmental* **2017**, 218, 488−497.
3. Khobragade R.; Roškarič M.; Žerjav G.; Košiček M.; Zavašnik J.; Van de Velde N.; Jerman I.; Tušar N.N.; Pintar A. Exploring the effect of morphology and surface properties of nanoshaped Pd/CeO_2 catalysts on CO_2 hydrogenation to methanol. *Applied Catalysis A: General* **2021**, 627.
4. Bahruji H.; Bowker M.; Hutchings G.; Dimitratos N.; Wells P.; Gibson E.; Jones W.; Brookes C.; Morgan D.; Lalev G. Pd/ZnO catalysts for direct CO_2 hydrogenation to methanol. *Journal of Catalysis* **2016**, 343, 133−146.

5. Lawes N.; Aggett K.J.; Smith L.R.; Slater T.J.A.; Dearg M.; Morgan D.J.; Dummer N.F.; Taylor S.H.; Hutchings G.J.; Bowker M. Zn Loading Effects on the Selectivity of PdZn Catalysts for CO_2 Hydrogenation to Methanol. *Catalysis Letters* **2023**, 154, 1603−1610.
6. Pan H.; Ma B.; Zhou L.; Hu Y.; Shakouri M.; Guo Y.; Liu X.; Wang Y. Highly Efficient CuPd0.1/γ−Al_2O_3 Catalyst with Isolated Pd Species for CO_2 Hydrogenation to Methanol. *ACS Sustainable Chemistry & Engineering* **2023**, 11, 7489−7499.
7. Wang X.; Yang X.; Pei G.; Yang J.; Liu J.; Zhao F.; Jin F.; Jiang W.; Ben H.; Zhang L. Strong metal–support interaction boosts the electrocatalytic hydrogen evolution capability of Ru nanoparticles supported on titanium nitride. *Carbon Energy* **2023**, 6.
8. Song J.; Liu S.; Yang C.; Wang G.; Tian H.; Zhao Z.−j.; Mu R.; Gong J. The role of Al doping in Pd/ZnO catalyst for CO_2 hydrogenation to methanol. *Applied Catalysis B: Environmental* **2020**, 263.
9. Jiang X.; Koizumi N.; Guo X.; Song C. Bimetallic Pd–Cu catalysts for selective CO_2 hydrogenation to methanol. *Applied Catalysis B: Environmental* **2015**, 170−171, 173−185.

Table S3. The assignment of *in−situ* DRIFT peaks.

Sample	InO_x	0.4Pd−InO_x	1.2Pd−InO_x
Area of *CO (2026 cm^{-1})	0.00716	0.00808	0.00754
Area of *HCOO (1559 cm^{-1})	0.01653	0.00961	0.00516
*CO /*HCOO	3/7	5/6	11/2
